# Assessment of Type 2 Diabetes Awareness and Knowledge in the Non-medical Bahraini Population

**DOI:** 10.7759/cureus.44231

**Published:** 2023-08-28

**Authors:** Saeed H Khalaf, Aysha S Waheed, Noora A Ali, Noor J AlNajem, Rawan M Abdulrahman, Zainab J Hasan

**Affiliations:** 1 Endocrinology, Salmaniya Medical Complex, Manama, BHR; 2 General Practice, Royal College of Surgeons in Ireland, Busaiteen, BHR

**Keywords:** diabetes mellitis, bahrain, type 2 diabetes mellitus, general population, awareness

## Abstract

Background

With type 2 diabetes (T2D) posing an escalating health challenge to the Kingdom of Bahrain, enhancing public awareness becomes instrumental in mitigating disease outcomes. This study aimed to appraise the level of T2D awareness among the non-medical Bahraini population by evaluating their understanding of the disease, its risk factors, symptoms, complications, monitoring, and prevention methods.

Methods

We conducted a cross-sectional study from March 2022 to June 2022. The study employed an electronic survey using Google® Forms (Google LLC, Mountain View, California, USA) targeting non-healthcare workers aged 15 and above. The survey consisted of multiple yes-and-no questions designed to evaluate different aspects of respondents’ T2D knowledge.

Results

Out of 835 participants, a total of 613 participants were included. The overall awareness of T2D was found to be average (70.6% CI±6.214, SD ±8.80%). The mean scores of correct answers in the different sections such as general knowledge, risk factors, symptoms, complications, treatment, monitoring, and prevention of T2D were 54.5% CI ±14.8, 75.5% CI±12.5, 77.6% CI±5.3, 61.8% CI±8.5, and 73.4% CI±5.4 respectively. Furthermore, the diabetic population scored an average of 76.7% in awareness in comparison to the non-diabetic population which scored 72.5% in overall awareness.

Conclusions

These findings underscore the pressing need to enhance T2D awareness among the Bahraini population. Implementing early education programs and strategically utilizing social media platforms may aid in bolstering public understanding of T2D, aiming ultimately to reduce its prevalence and associated economic burden.

## Introduction

Type 2 diabetes (T2D) poses a mounting health challenge in the Kingdom of Bahrain. As of 2022, the prevalence of diabetes in Bahrain stood at 9.0%, with an incidence rate of 14.7% [1,2]. This represents a considerable national and international health concern [3,4]. The total expenditure associated with diabetes-related health issues in the nation was projected at 158.7 million USD in 2021 [3]. Moreover, diabetes often increases morbidity and mortality rates due to macrovascular complications such as diabetic neuropathy, diabetic nephropathy, and diabetic retinopathy [5,6]. Various studies underscore the necessity for public health programs to mitigate this burden [5,7,8]. The Ministry of Health in Bahrain, alongside various support groups, has made significant efforts to raise public awareness about diabetes and its complications, employing strategies like awareness campaigns, staff education and training, and the establishment of diabetes clinics [2]. This study seeks to evaluate the awareness and general understanding of T2D among the non-medical Bahraini population, focusing on disease prevention and monitoring knowledge.

This article was previously presented as an abstract at the 5th Bahrain Diabetes and Endocrine Review Conference on February 16-17, 2023.

## Materials and methods

We conducted a cross-sectional study at Salmaniya Medical Complex (SMC) from March 2022 to June 2022. The SMC Institutional Review Board from the Research Committee for Government Hospitals approved the study (serial number 39280322). We collected data via a modified electronic questionnaire [9,10] comprising 35 yes/no/unsure questions covering various topics, including demographic data, general knowledge of diabetes, risk factors, symptoms, complications, treatment, monitoring tests, and prevention methods. The questionnaire was disseminated through social media platforms and to those visiting the hospital during the study period. All participants provided voluntary informed consent prior to participating in the study. The inclusion criteria included anyone over the age of 15 with access to social media who could read Arabic or English. However, the exclusion criteria included healthcare workers and those who did not meet the inclusion criteria.

Data was collected in Google® Forms (Google LLC, Mountain View, California, USA) and later was analyzed by Microsoft Excel 2010 (Microsoft Corp., Redmond, WA, USA) to calculate the sum and percentage of each question. Furthermore, data was reported as mean (standard deviation) and percentages.

## Results

Study demographics

We received 835 completed questionnaires. We excluded a total of 222 responses, from which 215 were healthcare workers and 7 respondents did not give consent. The study included 613 respondents in the final analysis, with 106 (17.3%) respondents diagnosed with diabetes.

Table 1 reveals that 411 (67%) participants were female and 202 (33%) were male. Participants ranged in age from 15 to over 60, most within the 15- to 30-year-old category with 275 (44.8%) participants. Most participants (n=453; 73%) held university degrees. Additionally, 468 (76.3%) participants had at least one relative with diabetes, while 145 (23.7%) had no family history of the disease. The scores of this study represent the population’s level of knowledge about T2D, with higher scores indicating greater knowledge.

**Table 1:** Study population characteristics

Category		N (%)
Sex	Female	411 (67%)
	Male	202 (33%)
Age group	15-30 years	275 (44.8%)
	31-45 years	178 (29%)
	46-60 years	120 (19.6%)
	Above 60 years	40 (6.5%)
Educational level	University graduates	453 (73.9%)
	High school graduates	148 (24.1%)
	Secondary school graduates	10 (1.6%)
	Primary school graduates	2 (0.3%)
History of diabetes	No	507 (82.7%)
	Yes	106 (17.3%)
Family member with diabetes	Yes	468 (76.3%)
	No	145 (23.7%)

Overall awareness among different age groups and education levels

Analysis of the average scores of different age groups revealed that participants aged 31-45 demonstrated a higher overall awareness level, with an average score of 76.99%. This compared to the 15-30 years, 46- 60 years, and over 60 years age groups, who scored averages of 70.76%, 75.25%, and 67.69%, respectively. Regarding education levels, secondary school graduates displayed the highest level of awareness, with an average score of 78.79%. This compares to scores of primary graduates (36.66%), high school graduates (71.86%), and university graduates (73.71%).

Knowledge about T2D

The general knowledge section on T2D registered the lowest average score, with only 54.5% (CI ±14.8) answering correctly. A total of 349 (56.9%) participants correctly identified diabetes as a condition of high blood glucose (i.e., “sugar”), while 280 (45.7%) acknowledged it as a condition of insufficient insulin in the blood. Furthermore, 204 (33.3%) correctly identified diabetes as a condition where the body does not respond to insulin, and 492 (80.2%) correctly classified it as a noncontagious disease. However, only 185 (30.2%) participants understood that T2D is incurable (Table 2).

**Table 2:** Participants’ awareness of T2D T2D: type 2 diabetes, BMI: body mass index

Diabetes information	Responses, n (%)		
	Correct	Wrong	Unsure
T2D general knowledge			
Diabetes is a condition of high blood sugar	349 (56.9%)	63 (10.3%)	201 (32.8%)
Diabetes is a condition of not having enough insulin in the blood	280 (45.7%)	98 (16%)	235 (38.3%)
T2D is a condition of the body not responding to insulin	204 (33.3%)	141 (23%)	268 (43.7%)
T2D is not curable	185 (30.2)	218 (35.5%)	210 (34.3%)
T2D is contagious	492 (80.2%)	23 (3.8%)	98 (16%)
T2D occurs in children, adolescents, and adults	344 (56.1%)	95 (15.5%)	174 (28.4%)
Insulin is a hormone that controls blood sugar levels	487 (79.4%)	35 (5.7%)	91 (14.8)
T2D risk factors			
Family history of diabetes	470 (76.7%)	50 (8.2%)	93 (15.1%)
Age above 40 years	394 (64.3%)	86 (14%)	133 (21.7%)
Obesity	525 (85.6%)	27 (4.4%)	61 (10%)
T2D symptoms			
Constant feeling of thirst	435 (70.96%)	46 (7.5%)	132 (21.53%)
Increased frequency of urination, especially at night	507 (82.7%)	25 (4%)	81 (13.2%)
Blurred vision	463 (75.53%)	33 (5.38%)	117 (19%)
Poor healing of cuts and wounds	498 (81.23%)	25 (4%)	90 (14.68%)
T2D complications			
Eye problems	469 (76.5%)	32 (5.22%)	112 (18.27%)
Kidney problems	379 (61.82%)	62 (10.11%)	172 (28%)
High blood pressure	311 (50.7%)	85 (13.86%	217 (35.39%
Loss of sensation in arms and legs	343 (55.95%)	58 (9.46%)	212 (34.58%)
Decaying of limbs that require amputation	394 (64.27%)	61 (9.95%)	158 (25.77%)
Are these T2D treatments?			
Tablets and capsules to control diabetes	433 (70.6%)	65 (10.6%)	115 (18.8%)
Insulin injections to control diabetes	467 (76.1%)	61 (10%)	85 (13.9%)
Monitoring T2D			
Monitoring blood sugar levels	574 (93.63%)	8 (1.3%)	31 (5%)
Routine eye check-ups	489 (79.77%)	30 (4.89%)	94 (15.33%)
Routine check-ups	549 (89.55%)	9 (1.46%)	55 (8.97%)
T2D prevention			
Eating healthy food and avoiding sweets and sugary snacks	555 (90.53%)	20 (3.26%)	38 (6.19%)
Eating less, skipping meals, and starving	347 (56.6%)	185 (30.1%)	81 (13.21%)
Exercising on a regular basis	577 (94.12%)	8 (1.3%)	28 (4.56%)
Maintaining BMI within a healthy range	549 (89.55%)	14 (2.28%)	50 (8.15%)
Reducing stress levels	492 (80.26%)	32 (5.22%)	89 (14.51%)

Knowledge of T2D risk factors

The majority of participants (75.5% CI±12.5) were aware of obesity and family history as risk factors for T2D, demonstrated by 525 (85.6%) and 470 (76.7%) correct responses, respectively. However, fewer participants, 394 (64.3%), recognized age as a risk factor.

Knowledge of T2D symptoms and complications

The majority of participants correctly identified increased thirst, increased urination, blurred vision, and poor healing of cuts and wounds as symptoms of T2D: 435 (70.9%), 507 (82.7%), 463 (75.5%), and 498 (81.2%), respectively. Participants had a mean score of 61.8% (CI±8.5) regarding knowledge of T2D complications. While 469 (76.5%) recognized “eye problems” as a complication of T2D, fewer were aware of other complications such as “loss of sensation” (n=343; 56%), “high blood pressure” (n=311; 50.7%), “kidney problems” (n=234; 38.2%), and “decay of limbs” (n=219; 35.7%).

Knowledge of T2D treatment and monitoring

On average, 73.4% (CI±5.4) of participants correctly identified current T2D treatments, including tablets and insulin injections. Knowledge about monitoring strategies was robust, with an average of 87.7% (CI±8.1) correct answers. These strategies included monitoring blood sugar (n=574; 93.6%), routine eye examinations (n=489; 79.77%), and regular checkups (n=549; 89.55%).

Knowledge about T2D prevention

Participants scored well in this section, averaging 87.0% (CI±13.3). The majority correctly identified exercise (n=577; 94.1%), a healthy diet (n=555; 90.5%), maintaining a healthy body mass index (n=549; 89.5%), and reducing stress levels (n=492; 80.3%) as preventative measures. However, there was some confusion about whether eating less, skipping meals, and starving were good preventative methods, with only 347 (56.6%) correctly refuting this assertion.

Overall awareness in the general, diabetic, and non-diabetic population

**Figure 1:**
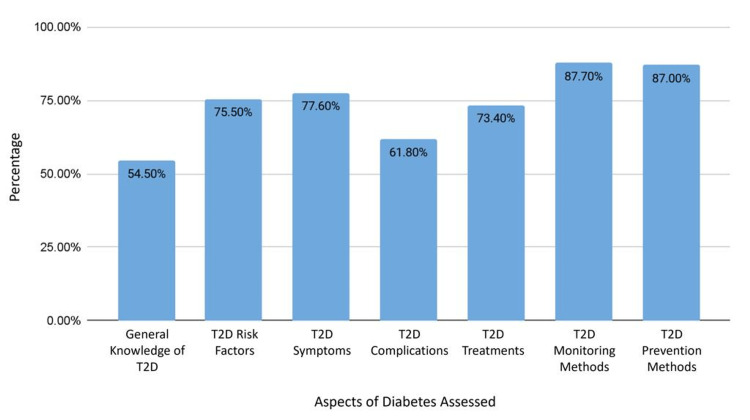
The average scores of awareness in the general population T2D: type 2 diabetes

**Figure 2:**
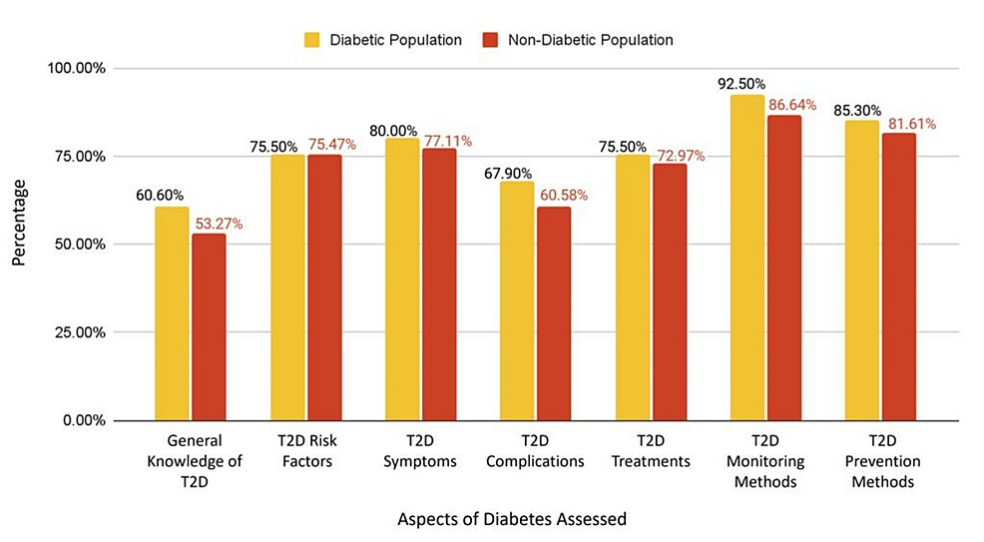
The average scores of awareness in the diabetic and non-diabetic population T2D: type 2 diabetes

The general population’s overall T2D awareness averaged 70.6% (CI ±6.214, SD ±8.80%), with a detailed breakdown in Figure 1. The diabetic population displayed a higher average awareness of 76.7%, compared to the non-diabetic population, which averaged 72.5%. Figure 2 compares the average scores of the diabetic and non-diabetic populations across each section of the study.

## Discussion

Our study sought to evaluate the understanding of T2D among the non-medical Bahraini population. We observed an overall T2D awareness average of 70.6%. Notably, among participants diagnosed with diabetes, the awareness rate increased slightly to 76.7%. We anticipated a higher awareness level among diabetic participants due to the T2D education they presumably received from their general practitioners or endocrinologists upon diagnosis [2]. However, the minor difference suggests that these individuals may lack comprehensive disease understanding, which could impact their treatment adherence and future health outcomes.

Despite various awareness-raising efforts implemented in Bahrain [2], no previous study has gauged their effectiveness in improving public T2D knowledge. A 2003 study assessing T2D awareness among Bahraini teachers reported a rate of 53.4% [11]. Although our study indicates a significant increase, a direct comparison remains challenging due to the previous study’s focus on teachers who had received diabetes education. Moreover, our study’s overall awareness level (70.6%) exceeded that found in similar research conducted in Kuwait (63.2%) and Singapore (67.3%) [9,10]. The awareness campaigns launched by the Ministry of Health and various Bahraini support groups since 2003 may have contributed to this increase [2].

The low general knowledge scores about T2D in both the general (54.5%) and diabetic populations (60.6%) are concerning. A number of participants believed T2D was curable (35.5%), a misunderstanding echoed in other studies [10,12]. Further, 6.6% of diabetic participants believed T2D was contagious, underlining the importance of enhancing patient education about the disease’s nature and pathophysiology to promote treatment adherence and improve prognosis.

When considering T2D risk factors, many participants identified obesity (85.6%) and less so family history (76.7%) and age (64.3%). As obesity is a modifiable risk factor [12], its recognition could be attributed to the more extensive discussion by physicians, particularly during preventative measure counseling. Given the sedentary lifestyle and high-fat diet common among Bahrainis [13], a greater understanding of the risk factors associated with T2D is crucial.

Concerningly, only 30.6% of participants could identify all T2D complications. A 2019 Bahraini study reported that nearly half of diabetic patients suffered from at least one complication [14]. However, our participants were largely unaware that T2D could lead to loss of sensation or high blood pressure. In contrast, a study in Saudi Arabia reported an 80% awareness of T2D complications among their population [15].

Concerning T2D treatment, over half of the participants were aware of the different treatments, notably insulin. This recognition likely stems from the high prevalence of uncontrolled diabetes in Bahrain [2]. Indeed, a 2019 Bahraini study reported that nearly a quarter (23.6%) of T2D patients were receiving insulin therapy [14].

The highest accuracy rates were found in the sections concerning T2D monitoring and prevention. This finding may reflect the effective primary healthcare practice in Bahrain, where specialized diabetic clinics in each healthcare center provide routine checkups for diabetic patients [2]. However, it is concerning that 30.1% of respondents, including 50.9% of patients diagnosed with diabetes, believed that skipping meals or starving could prevent diabetes. This misconception highlights the need for education about maintaining a healthy diet, as inadequate meal intake can lead to hypoglycemia and other serious issues [16].

Several potential limitations in this study should be acknowledged. The absence of previous studies on this topic in Bahrain limits the scope of the literature review and historical comparison. The online questionnaire may also restrict the participant pool due to limited electronic device access. Future studies should consider expanding the sample size for more representative results.

## Conclusions

Our study sought to ascertain the level of awareness regarding T2D in Bahrain’s non-medical population. Our findings highlight that a significant portion of the population exhibits a solid understanding of T2D, although there is room for improvement. This underscores the necessity for enhanced efforts to elevate public understanding of T2D to mitigate the incidence of the disease and its associated economic burden. Key strategies could include early education initiatives targeted at school students and leveraging social media platforms for broader reach. In the long run, improving public awareness and routinely assessing their knowledge could reduce the prevalence of T2D and its economic impact.
